# Visual Mislocalization of Moving Objects in an Audiovisual Event

**DOI:** 10.1371/journal.pone.0154147

**Published:** 2016-04-25

**Authors:** Yousuke Kawachi

**Affiliations:** Kansei Fukushi Research Institute, Tohoku Fukushi University, Sendai, Japan; University of Verona, ITALY

## Abstract

The present study investigated the influence of an auditory tone on the localization of visual objects in the stream/bounce display (SBD). In this display, two identical visual objects move toward each other, overlap, and then return to their original positions. These objects can be perceived as either streaming through or bouncing off each other. In this study, the closest distance between object centers on opposing trajectories and tone presentation timing (none, 0 ms, ± 90 ms, and ± 390 ms relative to the instant for the closest distance) were manipulated. Observers were asked to judge whether the two objects overlapped with each other and whether the objects appeared to stream through, bounce off each other, or reverse their direction of motion. A tone presented at or around the instant of the objects’ closest distance biased judgments toward “non-overlapping,” and observers overestimated the physical distance between objects. A similar bias toward direction change judgments (bounce and reverse, not stream judgments) was also observed, which was always stronger than the non-overlapping bias. Thus, these two types of judgments were not always identical. Moreover, another experiment showed that it was unlikely that this observed mislocalization could be explained by other previously known mislocalization phenomena (i.e., representational momentum, the Fröhlich effect, and a turn-point shift). These findings indicate a new example of crossmodal mislocalization, which can be obtained without temporal offsets between audiovisual stimuli. The mislocalization effect is also specific to a more complex stimulus configuration of objects on opposing trajectories, with a tone that is presented simultaneously. The present study promotes an understanding of relatively complex audiovisual interactions beyond simple one-to-one audiovisual stimuli used in previous studies.

## Introduction

The perceptual system estimates spatial positions, relationships, and trajectories of moving objects to understand causal relations [1], social interactions [2], and situations with obscured object locations. For a precise estimation, the system accumulates available sensory inputs to different modalities from events, across space and time. However, perceived positions and trajectories are not always veridical and are flexibly modulated by interactions between multisensory inputs.

Many previous multisensory studies have reported that vision provides precise spatial information to dominate a spatial percept, while audition provides precise temporal information. It has been assumed that multisensory interactions are subject to a ‘‘Modality Appropriateness” hypothesis [3], which postulates that a sensory modality more appropriate to a given task has a dominant effect on performance. A representative example is the spatial ventriloquism effect [4]. For this effect, the location of auditory stimuli (e.g., the ventriloquist’s speech without lip movements) is mislocalized toward the location of the visual stimulus that is presented simultaneously with the auditory one (e.g., the puppet’s lip movements). However, there are several reports suggesting that auditory information can affect visual spatial processing: spatial distortion mediated by auditory information attracting the perceived timing of a visual event [5–9]. An auditory tone presented before or after a visual flash can draw the perceived timing of the flash towards that of the auditory stimulus. This phenomenon is known as “temporal ventriloquism.” In previous studies [5, 6], temporal ventriloquism has an effect on the performance of a visuospatial task in which observers judge the spatial position of a flash relative to that of a moving object (e.g., flash-lag effect paradigm). An auditory stimulus is presented temporally proximal to a flash and modulates perceived timing of the flash, which results in the modulation of the perceived spatial relationship between the flash and the moving object. Furthermore, Heron et al. [7] reported that a tone presented prior to the turn of a moving object causes the turn-point to be mislocalized in front of the physical turn-point. Non-simultaneity of audiovisual stimuli seems to be critical for such displacement phenomena. Teramoto, Hidaka, Gyoba and Suzuki [8] and Chien, Ono, and Watanabe [9] showed that auditory sustained or transient stimulation modulates the perceived disappearing point of a moving object. Auditory offset after or before the physical disappearance biases the perceived disappearing point in the forward or backward direction of visual motion, respectively. Thus, although visual mislocalization by an auditory stimulus is apparently inconsistent with visual dominance in the spatial domain, these studies [5–9] suggested that the auditory modulation of visual localization needs an auditory stimulus to attract the timing of a visual stimulus toward the auditory one.

The present study demonstrates a new type of audiovisual mislocalization in which it is unnecessary for an auditory stimulus to modulate perceived visual stimulus timing (see [10], for a related study). We used an audiovisual stream/bounce display. The stream/bounce display (SBD) includes two visually identical objects, which move toward each other, and then move apart in a two-dimensional display. Observers either perceive the objects as streaming through or bouncing off each other. Although the SBD is ambiguous, many observers tend to perceive the two objects as streaming through each other [11, 12]. However, Sekuler, Sekuler and Lau [13] reported that a brief tone presented at the moment the two objects completely overlap (or come closest to each other) predominantly induces the bouncing percept. Thus, in general, previous studies using the SBD focused on perceived motion trajectories affected by an auditory tone. Interestingly, in our previous studies [14, 15], observers informally reported that visual objects do not appear to be overlapping when an auditory tone is presented at the moment the objects physically overlap. Based on participants’ introspective reports, we hypothesized that object overlap judgments would be affected by the auditory tone presented simultaneously with the overlapping of visual objects. This hypothesis is inconsistent with the modality appropriateness hypothesis. Given that the present task is not in the temporal domain, and audition does not have temporal information for attracting the perceived timing of a visual event, vision should dominate spatial localization performance. In the ambiguous stimulus configuration of the SBD, the perceptual system would have difficulty judging whether a sensory modality is appropriate and reliable for generating a veridical perception. Therefore, a visual dominance effect would not be sufficiently obtained. We expected that the perceptual system is able to utilize such a salient stimulus as a sudden auditory tone in a cluttered surrounding environment, to resolve perceptual ambiguity in temporal proximity with the tone [16].

The present study assessed whether the spatial positions of overlapping visual objects in the SBD could be mislocalized due to simultaneous presentation of an auditory stimulus, so as to be perceived as non-overlapping (Experiment 1). Although previous studies reported that the non-simultaneity of audiovisual stimuli generates the mislocalization of an object [7–9], it is important to report that physically near-simultaneous stimuli can generate object mislocalization. Typically, multiple near-simultaneous sensory inputs should be considered as generated by a physical event in the environment. Simultaneous audiovisual stimuli exclude the possibility that an auditory tone dominates the perceived timing of a visual stimulus to modulate visuospatial localization. Thus, we can show a new type of audiovisual mislocalization without the auditory dominance effect in the temporal domain. For the present study, the closest distance between object centers, that is, the amount of overlap between objects in the SBD, and the tone presentation (presence or absence) were manipulated. Observers were asked to judge whether the two objects appeared to overlap with each other. We predicted that this crossmodal mislocalization would suggest that visual dominance is degraded in the SBD and object localization is flexibly modulated through multisensory interaction triggered by a sudden auditory tone.

We also tested whether mislocalization occurred through an auditory stimulus presentation before and after the instant when the object centers were closest (Experiment 2). If temporal offsets between an auditory stimulus and the overlap of visual objects are needed for any observed mislocalization, the degree of mislocalization in non-zero offset conditions may be larger than in the zero offset (simultaneity) condition, in line with studies of similar audiovisual effects [7–9]. This temporal profile is considered one of the main properties of visuospatial processing and crossmodal interaction. Object localization does not seem to be immediate and requires some time. The perceptual system accumulates evidence of multisensory inputs over a spatial and temporal window and determines momentary visual representations. When a sound is presented before the visual event (i.e., before overlap), the system makes a prediction in which it expects a certain visual event (e.g., non-overlap of objects) on the basis of prior information (auditory tone before the overlap). When a sound is presented after the visual event it may make a postdiction in which it interprets the event by incorporating information obtained after the events (auditory tone after the overlap) [17]. This test will reveal those predictive/postdictive properties involved in mislocalization and help relate the present findings to previous ones, which were obtained with non-simultaneous audiovisual stimuli. Moreover, to further understand mislocalization, the present study explored whether our observed mislocalization could be accounted for by the size and direction of other phenomena and the sensitivity to an auditory stimulus within other phenomena (i.e., representational momentum [18], the Fröhlich effect [19, 20], and a turn-point shift [10]) (Experiment 3).

In addition to the overlap judgment task as described above, we asked participants to judge whether the two objects appeared to be streaming through, bouncing off each other, or reversing their direction of motion in Experiments 1 and 2. We elucidated how directional change judgments are related to the object overlap judgments in order to show that both judgments are not always consistent with each other. Although the relationship between both judgments has yet to be sufficiently examined, object localization reflected in overlap judgments seems to be closely related to the directional change judgment. This is because the closest points between object centers are the points where objects are perceived as changing their motion trajectories.

We tried to show a new type of crossmodal mislocalization in the SBD. Crossmodal mislocalizations are induced by an auditory stimulus, near simultaneous with visual stimuli, different from a non-simultaneous stimulus in previous studies (Experiment 1 and Experiment 2). This study also examined whether judgments regarding spatial positions (i.e., overlap judgments) and moving trajectories (i.e., directional change judgments) differ in terms of the SBD. Furthermore, crossmodal mislocalization is not predicted by previously reported mislocalization phenomena (Experiment 3). Our goal was to determine whether the present findings reveal a new crossmodal interaction inconsistent with the modality appropriateness hypothesis. During this interaction, vision does not dominate the visuospatial localization of objects, and a non-simultaneous auditory tone does not attract the timing of a visual event (auditory dominance effect in the temporal domain).

## Experiment 1

### Ethics statement

All experiments were approved by the Ethical Committee of Tohoku Fukushi University and conducted according to principles from the Declaration of Helsinki. Written informed consent was obtained from all participants.

### Participants

Nineteen healthy adults (4 males and 15 females) participated in the experiment. Their average age was 21.94 years (age range, 19–32 years). Except for one author (YK), all participants were naive as to the purpose of the experiment. All were right-handed, had either normal or corrected-to-normal vision, and reported no hearing anomalies.

### Stimuli

**Fig 1:**
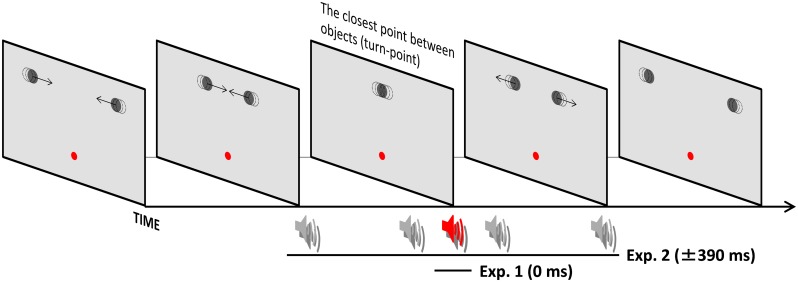
Schematic illustration of stimuli for Experiment 1. The objects moved from opposite sides toward each other. After 630 ms of motion, objects started to reverse their direction with some degree of overlap and returned to their original positions. The starting points for either object (dashed-line circle) were displaced to manipulate the distance between the object centers, that is, the degree of object overlap in a trial-by-trial manner.

All stimuli were generated on a PC (Dell Precision T5500; Dell: Austin, TX) running Matlab 2007a (The MathWorks, Inc.: Natick, MA) using the Cogent Graphics package (http://www.vislab.ucl.ac.uk/cogent.php). Visual stimuli were presented on a CRT monitor (Sony GDM-F520, Sony: Tokyo, Japan; refresh rate 100 Hz; resolution 1024×768 pixels). As shown in Fig 1, a red fixation dot was presented at the center of the monitor (13.19 cd/m2, 20.46 arc min of visual angle) on a gray background (52.99 cd/m2). Each one of the two objects subtended 36.37 arcmin of visual angle (13.33 cd/m2). The objects, presented 182.40 arcmin above the fixation point, moved from opposite sides toward each other. The monitor refresh rate was 100 Hz, but the actual frame rate of the animation was 33 frames per second (1 frame = 30 ms). Object positions were displaced every 30 ms. This displacement, combined with the frame rate, resulted in an object speed of 3.79 deg/s. After 630 ms of motion, objects started to reverse their direction with some degree of object overlap and returned to their original positions. The closest distance between object centers, that is, the amount of overlap between objects, was manipulated by increasing the inter-object distances: 0 (complete overlap), 13.64, 20.46, 27.28, 34.10, 40.92, and 54.56 arcmin between the object centers. Moreover, the spatial positions where the objects change motion trajectories fluctuated by the objects’ starting point jittering in the range of ± 61.38 arcmin on a trial-by-trial basis. In half of the trials, a brief tone (800 Hz, 10 ms, 68 dB SPL; background noise level: 40 dB) was introduced through headphones (HDA 200; Sennheiser: Wedemark, Germany), only at the instant for the closest distance. The physical simultaneity of auditory and visual stimuli was assessed with an oscilloscope (TBS 1102; Tektronix, Pittsfield, MA). Deviations from simultaneity did not exceed 5 ms.

### Procedure

Each participant sat approximately 57 cm away from the display and used a chin rest to stabilize the visual field. Observers judged whether two moving objects appeared to overlap and then judged whether the objects appeared to stream through, bounce off each other, or reverse their motion direction. Experimental conditions included the presentation of a brief tone (tone/no-tone) and the degree of maximal overlap between the objects (0, 13.64, 20.46, 27.28, 34.10, 40.92, or 54.56 arcmin). Each condition combination was repeated over 20 trials.

### Results

**Fig 2:**
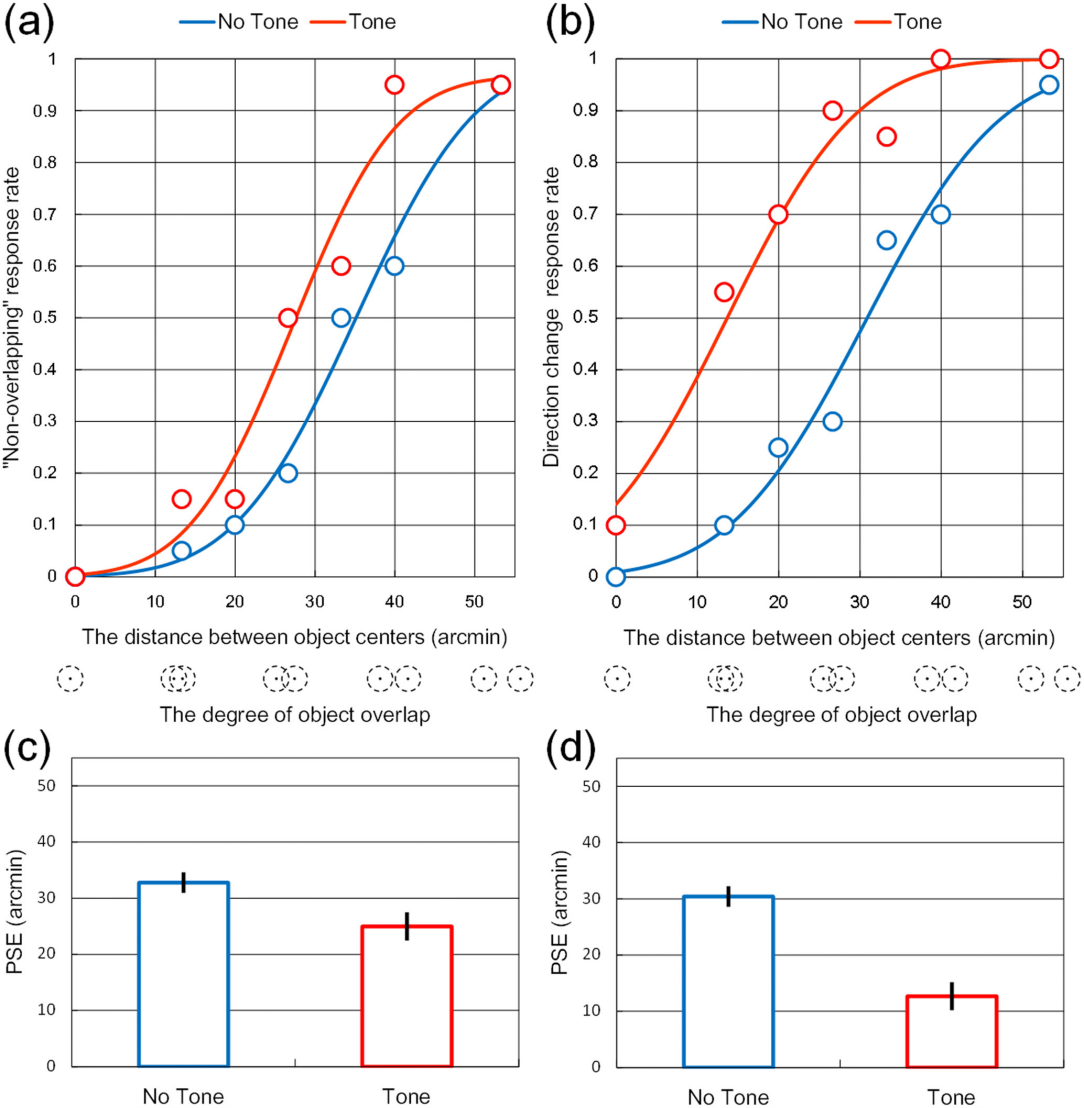
Results of Experiment 1. The psychometric functions fitted to “non-overlapping” judgment data (a) and directional change judgment data (b) from Subject 8. Below the abscissae, the degree of object overlap corresponds to the distance between object centers. Group mean PSEs for tone conditions in the (c) overlap and (d) directional change judgment tasks. Error bars denote standard errors of the mean (*n* = 19).

For the motion direction (stream/bounce/reverse) judgment task, we combined bounce and reverse judgments into directional change judgments. Then, a cumulative Gaussian function was fitted to each proportion of overlap judgments and directional change judgments (Fig 2a and 2b). We calculated the point of subjective equality (hereafter referred to as PSE) corresponding to 50% in the function (Fig 2c and 2d). In other words, the PSE indicates the closest distance between object centers (the degree of object overlap) at which point the participant is equally likely to report the objects as overlapping or not (for the overlap judgment task) or to report the objects as changing directions or not (for the motion direction judgment task) [21, 22]. For example, smaller (larger) PSEs mean that although objects overlap to a larger (smaller) extent, observers tend to perceive them as non-overlapping. A two-way repeated measures ANOVA for PSE was conducted with *judgment type* (2: overlap judgment task/motion direction judgment task) and *tone presentation* (2: tone/no tone) as factors. The main effects of judgment type and tone conditions were significant, *F*(1, 18) = 19.18, *p* < .001, and *F*(1, 18) = 79.44, *p* < .001. The interaction was also significant, *F*(1,18) = 9.23, *p* < .01. A post hoc analysis (Ryan’s method) revealed that the PSEs in the tone conditions were smaller than those in the no-tone condition, indicating that the tone influenced participants to underestimate the amount of overlap (i.e., overestimate the closest distance between object centers) and promoted directional change responses, *F*(1, 36) = 12.67, < .005, and *F*(1,36) = 66.31, < .001. Moreover, the PSE for the motion direction judgment task was smaller than for the overlap judgment task in the tone condition, *F*(1, 36) = 27.60, *p* < .001. Additionally, when objects completely overlapped, the direction change judgment rate was lower than what had been observed in several previous studies [13]. This low rate may be due to the relatively slow velocity of the object. Compared to the 6 deg/s velocity in Sekuler’s study, a 3.79 deg/s velocity is relatively slow. As object velocity increases, the bouncing response rate (i.e., direction change response rate) increases, irrespective of sound presentation [23]. Thus, our low direction change response rate may be due to a slower object velocity.

Finally, we conducted a one-sample *t*-test to examine whether there was a significant difference from 36.37 arcmin in terms of physical contact distances between objects (this value corresponds to object size) for the PSE during the overlap judgment task in the tone-present condition. Results showed that the PSE was smaller than the 36.37 arcmin of the physical contact distances, *t*(18) = -5.77, *p* < 0.001, indicating that participants illusorily overestimated the physical distance between the object centers.

We conducted a supplementary experiment with 13 healthy participants (9 new participants; 2 males and 11 females; average age of 21.92) in reversed task order. This was done in order to test the effect of motion direction judgment on the preceding overlap judgment task, as organized in the main experiment. Even in reversed task order (i.e., the motion direction judgment task after the overlap judgment task), almost identical results as those described above were obtained (PSEs for the overlap judgment: 34.77 arcmin in the no-tone condition, 27.74 arcmin in the tone condition; PSEs for the motion direction judgment: 30.43 arcmin in the no-tone condition, 15.50 arcmin in the tone condition). These results indicated that task order did not affect audiovisual mislocalization of objects.

## Experiment 2: Temporal Properties

Experiment 1 demonstrated an audiovisual mislocalization and a directional change bias induced by an auditory stimulus simultaneous with a visual event, with maximal object overlap. Meanwhile, several previous audiovisual mislocalization studies showed a maximal mislocalization at approximately ± 100 ms temporal offset between audiovisual signals [7–9]. Thus, we sought to demonstrate commonalities and differences between the present and previous studies by using non-simultaneous audiovisual stimuli. Moreover, this experiment explored whether the present audiovisual mislocalization could be predictively/postdictively obtained.

### Participants

Eleven healthy adults (3 males and 8 females) participated. Ten participants were from Experiment 1, and one of the authors (YK) participated. Average age was 22.27 years (age range, 19–32 years). Except for YK, all participants were naive as to the purpose of the experiment. All were right-handed, had either normal or corrected-to-normal vision, and reported no hearing anomalies.

### Stimuli and Procedure

The stimuli were identical to those in Experiment 1, except that sound conditions (no tone, ± 390 ms, ± 90 ms, and 0 ms offset relative to the maximal overlap point of the SBD) were used. The procedure for Experiment 2 was identical to that of Experiment 1.

### Results

**Fig 3:**
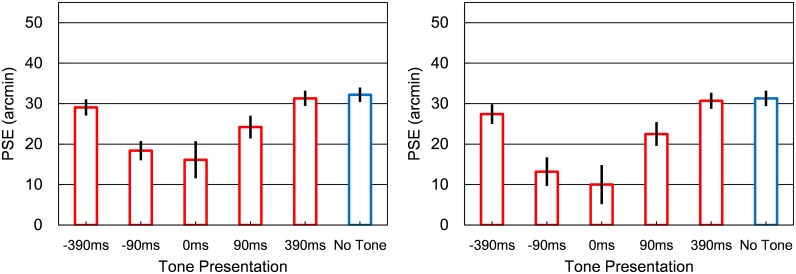
Results of Experiment 2. Group mean PSEs for tone conditions in the (a) overlap and (b) directional change judgment tasks. Error bars denote standard errors of the mean (*n* = 11).

For the motion direction (stream/bounce/reverse) judgment task, we combined bounce and reverse judgments into directional change judgments. Then, a cumulative Gaussian function was fitted to each proportion of overlap and directional change judgments. We calculated the PSE corresponding to 50% in the function (Fig 3a and 3b). A two-way repeated measures ANOVA for PSE was conducted with *judgment type* (2: overlap judgment task/motion direction judgment task) and *tone presentation* (6: no tone, ± 390 ms, ± 90 ms, and 0 ms offset) as factors. The main effects of judgment type and sound conditions were significant, *F*(1, 50) = 5.50, *p* < .05, and *F*(5, 50) = 15.81, *p* < .001, respectively. The interaction was also significant, *F*(5, 50) = 4.66, *p* < .005. A post hoc analysis (Ryan’s method) revealed that in the overlap judgment task, tone presentation significantly lessened the PSE in the −90 ms and 0 ms offset conditions compared to the no tone condition, *p*s < .05, indicating that a tone caused participants to underestimate the overlap amount. In the motion direction judgment task, tone presentation significantly lessened the PSE in the ± 90 ms and 0 ms offset conditions, *p*s < .05, indicating that a tone promoted the directional change response. Additionally, the PSEs were smaller in the −90 ms and 0 ms conditions compared to the 90 ms condition. Although the PSE in the ± 90 ms and 0 ms offset conditions was smaller than in the + 390 ms offset condition, only the PSE in the −90 ms and 0ms offset conditions was smaller than in the −390 ms offset condition. Moreover, compared to the overlap judgment task, PSEs at −90-ms and 0-ms offsets were smaller in the motion direction judgment task, *F*(1,60) = 11.65, < .005 and *F*(1,60) = 16.22, *p* < .001.

Finally, PSEs in the −90 ms, 0 ms, and 90 ms offset conditions were smaller than the 36.37 arcmin of the distances of physical contact, *t*(10) = −7.27, *p* < 0.001; *t*(10) = −6.36, *p* < 0.001; *t*(10) = −4.11, *p* < 0.005, with Bonferroni-corrections, indicating that participants illusorily overestimated the physical distance between the object centers (or underestimated the physical overlap). Meanwhile, in the ± 390 ms offset and no-tone conditions, there was no significant overestimation. Audiovisual mislocalization was obtained in the 0 and ± 90 ms offset conditions. However, the degree of mislocalization was not larger for certain temporal offsets between audiovisual stimuli compared to a zero offset, which was different from previous studies [7–9].

## Experiment 3: Crossmodal Modulation for Other Mislocalization Types

**Fig 4:**
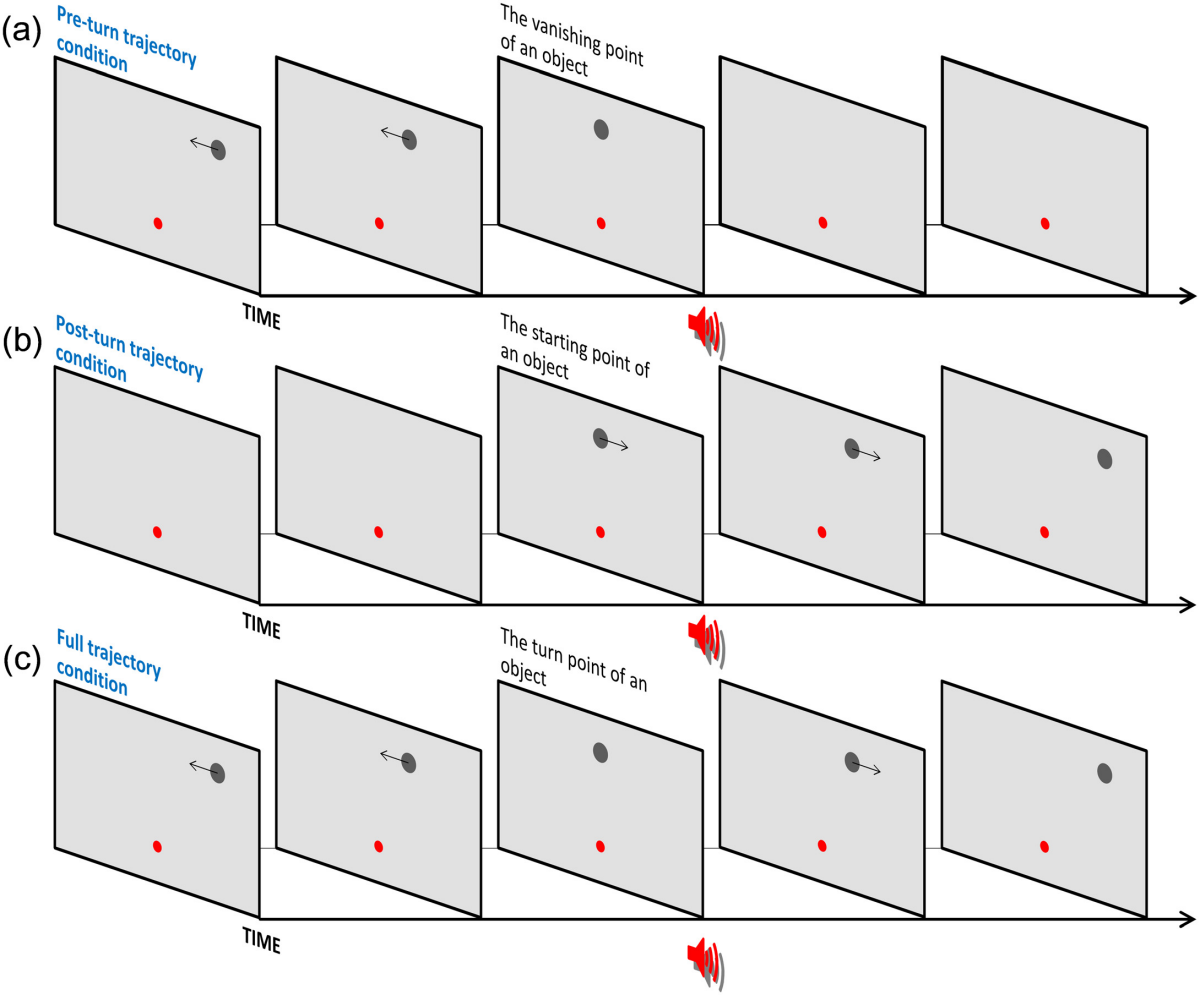
Schematic illustration of stimuli used in Experiment 3. (a) Pre-turn motion trajectory condition: the object moved proximal to the fixation point for 690 ms and then disappeared without the object turning. (b) Post-turn motion trajectory condition: the object appeared proximal to the fixation point, moved away from this location for 690 ms, and then disappeared. (c) Full motion trajectory condition: the object moved proximal to the fixation point for 690 ms, reversed its motion, and returned to the original position.

Experiments 1 and 2 demonstrated that a tone before and at the instant for the closest distance between object centers promoted the perception of “non-overlapping.” A tone also induced overestimation of the physical object distance for the 0 ms and ± 90 ms offset conditions. Experiment 3 explored the relationship between the observed audiovisual mislocalization and other mislocalization phenomena (i.e., representational momentum, RM; the Fröhlich effect, FE; onset repulsion, OR; or a turn-point shift, TPS). RM is where the vanishing point of a moving object is mislocalized further ahead in the motion direction [18]. FE (OR) refers to when the starting point of a moving object is mislocalized forward or backward in the motion trajectory [19, 20]. For the TPS, when a moving object moves diagonally and then suddenly turns 90 degrees, observers mislocalize the turn-point [10]. Observers tend to estimate the turn-point backward relative to the post-turn trajectory. In Experiment 3, we measured endpoint mislocalization of the pre-turn motion trajectory (RM), the starting point of the post-turn motion trajectory (FE), or the turn-point of the full motion trajectory (TPS) used in Experiments 1 and 2 (Fig 4). This was done in order to explore whether the present mislocalization could be associated with the endpoints and turn-point of the partial and full trajectory.

### Participants

Twelve healthy adults (2 males and 10 females) participated. Six participants were from Experiment 1, and one of the authors (YK) participated. Their average age was 21.84 years (age range, 20–35 years). Except for YK, all participants were naive as to the purpose of the experiment. All were right-handed, had either normal or corrected-to-normal vision, and reported no hearing anomalies.

### Stimuli and Procedure

The stimuli were identical to those from Experiment 1, except for the following: only one moving object was used, and an experimental block was conducted for each of the 3 motion trajectory conditions: (1) a pre-turn motion trajectory condition, in which the object moved to the left or right in proximity to the fixation point at 3.79 deg/s for 690 ms and then disappeared without a trajectory turn; (2) a post-turn motion trajectory condition, in which the object appeared proximal to the fixation point, moved away for 690 ms, and then disappeared; (3) a full motion trajectory condition, in which the object moved to the left or right in proximity to the fixation point for 690 ms. The object then reversed its motion and returned to its original position. In half of the trials, a brief tone was introduced through headphones and simultaneous with the endpoint (vanishing point) in the pre-turn motion trajectory condition, the starting point in the post-turn motion trajectory condition, and the turn-point in the full motion trajectory condition. Five hundred ms after observing the stimulus sequence, a probe object (identical to the moving object) was presented at ±36.38, ±9.09, and 0 arcmin relative to the endpoint in the pre-turn motion trajectory condition, the starting point in the post-turn motion trajectory condition, and the turn-point in the full motion trajectory condition. Positive (negative) values indicated forward (backward) positions of the vanishing point for the pre-turn trajectory. Observers judged whether the probe was located to the left or right, relative to the vanishing point, the starting point, and the turn-point. Each combination of tone presentation (tone/no-tone) and probe position (± 36.38, ± 9.09, or 0 arcmin) was repeated over 28 trials for each trajectory condition. Each block of 280 trials, which consisted of a block for each trajectory condition, was counterbalanced across participants.

### Results

**Fig 5:**
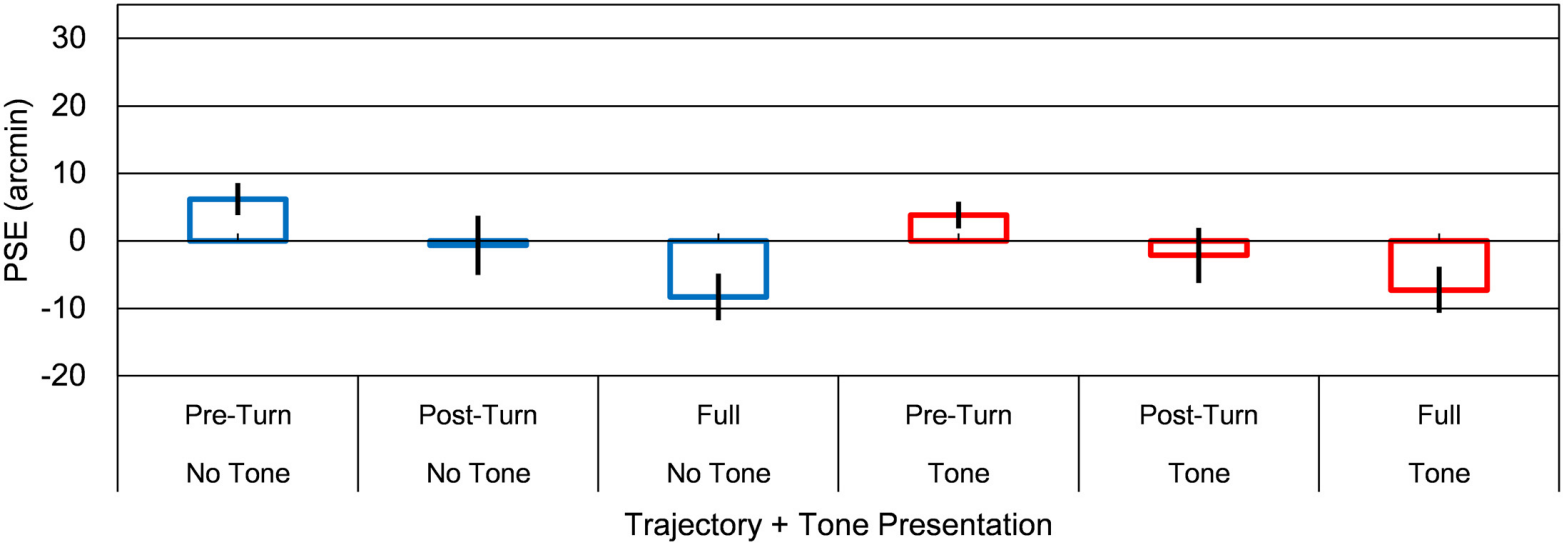
Results of Experiment 3. Group mean PSEs for tone conditions in combination with the 3 trajectory conditions. Positive (negative) PSEs indicate that the vanishing point, starting point, or turn-point was localized forward (backward) within the pre-turn trajectory, respectively. Error bars denote standard errors of the mean (*n* = 12).

A cumulative Gaussian function was fitted to each proportion of probe “right” judgments. The PSE was 50% in the function for the probe position to be perceived as being at a position equal to the vanishing point, the starting point, or turn-point (Fig 5). A two-way repeated measures ANOVA for PSE was conducted with *trajectory* (3: full, pre-turn, and post-turn motion trajectory conditions) and *tone presentation* (2: tone/no-tone) as factors. The main effect of trajectory condition was significant, *F*(2, 22) = 6.55, *p* < .01. The interaction was not significant, *F*(2, 22) = 1.26, *p* = .029. A post hoc analysis (Ryan’s method) revealed that mislocalization of the turn-point backward in the pre-turn trajectory was larger in the full motion trajectory condition than in the pre-turn trajectory condition (*p* < .05). Finally, we conducted one-sample *t-*tests with Bonferroni-corrections to examine whether the PSE was significantly different from the physical vanishing point, starting point, or turn-point. Results revealed no significant differences. These findings indicated that only a turning motion trajectory (full motion trajectory condition) promoted mislocalization of the turn-point backward (forward) in the pre-turn (post-turn) motion trajectory. Considering the turn-point as the starting point for the post-turn trajectory, mislocalization in the full trajectory condition is similar to the FE. However, the sizes of these mislocalizations were not significantly different from the physical zero (no mislocalization). Moreover, tone presentation did not modulate this mislocalization phenomenon. Accordingly, observed audiovisual mislocalization in the SBD was unlikely accounted for by other mislocalization phenomena.

## Discussion

The present study examined the influence of an auditory tone on the localization of visual objects embedded in the SBD. In Experiment 1, we demonstrated that a tone presented at the instant of a maximal overlap (i.e., the closest distance between objects) promoted perceptual non-overlap in the SBD. The degree of a perceived non-overlap was larger in the tone-present condition than in the no-tone condition, and it was also larger than that of the physical non-overlap. In other words, a tone that is simultaneous with a visual event (maximal overlap) induces spatial mislocalization of objects to repel each other. Previous studies showed that a tone presented before or after a visual event is necessary for the occurrence of mislocalization [7–9]. In these previous studies, the timing of a visual event may be attracted toward the timing of a tone, resulting in mislocalization of a visual event in the spatial domain. In those studies, audiovisual simultaneity generates little or no mislocalization. Thus, mislocalization observed in the current study provides a new example of crossmodal mislocalization that is not mediated by a tone attracting the timing of a visual event. The present mislocalization can now be added to the list of crossmodal phenomena that is clearly inconsistent with a ‘‘Modality Appropriateness” hypothesis. This hypothesis states that the sensory modality that provides more accurate information for a given task dominates performance. According to this hypothesis, vision should have dominated the overlap judgment (spatial localization judgment) in the present study. Audition also provided no temporal information for attracting the timing of a visual event toward that of the auditory stimulus. Moreover, several studies assessing crossmodal interactions have indicated that a concurrent auditory stimulus enhances perceived visual stimulus intensity [24, 25]. Nevertheless, the present findings can be interpreted such that a concurrent auditory stimulus interferes with the perception of visual objects at the closest distance, resulting in significant mislocalization of visual objects.

The present results also show commonalities and differences within the temporal domain, for the present and previous studies through Experiment 2, by using non-simultaneous audiovisual stimuli. Although only a tone in the 0 and −90 ms offset conditions induced a bias toward non-overlapping judgments compared to the no-tone condition, our observed audiovisual mislocalization was persistent across ± 90 ms temporal offsets between a tone and a to-be-modulated visual event (i.e., maximal overlap or the closest points between objects in the SBD). A tone presented before or after a to-be-modulated visual event predictively and postdictively modulates object localization [26]. Thus, our observed audiovisual mislocalization in the SBD had similar temporal characteristics to those of previous audiovisual mislocalizations. However, some temporal offset conditions did not generate a larger degree of mislocalization than a zero temporal offset (0 ms condition) in the present study. This differed from previous studies [7–9]. Therefore, the results of Experiment 2 more clearly support the notion that a tone that attracts the timing of a visual event is not necessary for mislocalization. A tone will directly affect the localization of visual moving objects. The results also suggest a temporal window in which the mislocalization effect is largest near the synchrony between audiovisual stimuli and becomes smaller as a temporal offset between audiovisual stimuli increases.

Here we will consider what occurs when moving objects overlap along crossing trajectories. Goldberg and Pomerantz [27] and Qian, Andersen, and Adelson [28] have stated that the motion system averages signals in opposite directions within a small region (similar to the overlapping of objects), yielding a zero resultant (null vector), which is assigned to the objects. Moreover, we have limited capacities for attentively tracking objects when they overlap or are very close to each other. At a maximal overlap, or the closest (non-overlapping) points of objects, participants may have difficulty distinguishing those objects. This loss of discriminability makes the attentive tracking of objects difficult [29]. The perceptual system has the complex task of gathering motion signals and/or tracking objects. Meanwhile, these weaker motion signals and/or object representations around the closest points are interpolated by motion signals integrated along with previous motion trajectories [23, 30]. As a result, the decision reached by the perceptual system may result in an overlap percept by default.

For the tone-present condition in this study, we need to consider the possible role of an auditory transient on weaker motion signals and/or object representations at the closest points of objects. If attention toward moving objects is distracted (i.e., attentional resources are captured) by a sudden tone when the objects were at the closest point, visual information processing may be temporally delayed [31]. Concretely, interpolating motion signals at the closest point may be delayed and interrupted for some time by shifting attention toward the auditory modality. Then, motion signals (around the closest point) that are re-processed and collected some time after an abrupt tone presentation may not be strong enough to be perceptible. This may generate a non-overlap percept in the SBD [15, 32]. Thus, the observed mislocalization suggests that weaker motion signals and/or object representations within a complex stimulus are paid less attention to. This is because presentation of an auditory transient distracts attention toward the auditory domain.

Interestingly, in Experiments 1 and 2, the PSEs in the tone-present condition for the overlap judgment task were significantly larger than for the motion direction judgment task. Moreover, PSEs for the overlap and motion direction judgment tasks are characterized by different temporal properties: for the motion direction judgment task, tone presentation at and before the instant of the closest distance has a stronger effect than a tone after the timing. Kawachi et al. [15] suggested that if a tone occurs before complete object overlap in the SBD, the observer can deploy attention toward the tone with some time left over for determining how the tone relates to the complete overlap. Alternatively, if the tone is presented too long after the complete overlap, by the time attention has been deployed toward the sound, the objects are too far apart to be interpreted as changing their motion direction (e.g., bouncing). Thus, the present study shows that whereas tone presentation induces participants to overestimate the closest distance (i.e., underestimate the degree of overlap), the overlap and motion direction judgments had different susceptibilities to the tone presentation. The motion direction judgment task (stream/bounce judgments in the conventional SBD study) is mainly influenced by response biases in the terminology referential to signal detection theory (SDT) [33]. Based on SDT, an overlap detection task is assumed to be affected by perceptual sensitivity and response biases [34]. Considered together, part of the stronger tone effect may be accounted for by a relatively strong response bias in the motion direction judgment task. These findings suggest that although both judgments may be partially supported by common perceptual processes, the overlap judgments are likely less susceptible to response biases compared to the direction change judgments. However, it should be noted that although a few studies indicated the effect of perceptual sensitivity/response biases on the SBD [33, 34], the detailed contents of each process remains to be elucidated. Recently, Witt, Raylor, Sugovic, and Wixted [35] pointed out that signal detection measures could not distinguish “perceptual” biases from response biases.

To further explore the observed mislocalization effects, Experiment 3 was conducted to relate the present findings to other mislocalization phenomena (i.e., FE, RM and TPS) and to explore a possible account for audiovisual mislocalization in the SBD. However, mislocalization of the endpoint of the pre-turn trajectory (RM), the starting point of the post-turn trajectory (FE), or the turn-point of the full trajectory (TPS) does not seem to contribute to the present phenomenon. Critically, we found that these mislocalizations were not modulated by tone presentation. Moreover, although the sizes and direction of these mislocalizations were significantly different from each other, the sizes of these mislocalizations were not significantly dissociable from the physical zero (no mislocalization). Therefore, it may be difficult to account for our mislocalizations by those associated with an endpoint and a turn-point within the partial and full trajectories. Thus, the results may point to a new type of mislocalization obtained from a relatively complex stimulus configuration: visual objects moving in opposite directions.

The present study clearly indicates that the spatial positions of moving objects on opposing trajectories are mislocalized by an auditory stimulus that is presented slightly before, at, or after the instant of their closest distance. However, slight temporal offsets between visual and auditory events do not promote the present mislocalization, as observed in previous studies. Larger temporal offsets do not have an effect on object localization. The present crossmodal mislocalization is inconsistent with the modality appropriateness hypothesis whereby vision dominates audition in the spatial domain [3]. This is an example of crossmodal mislocalization, which is not mediated by an auditory stimulus attracting the perceived timing of a visual stimulus. Even during a visuospatial task (i.e., overlap judgment task), the perceptual system may construct perceptual content that is strongly dependent on more reliable and salient sensory information. Moreover, auditory stimulation may not affect possible mislocalizations observed at the endpoint of the pre-turn trajectory (RM), the starting point of the post-turn trajectory (FE), or the turn-point of the full trajectory (TPS). Therefore, mislocalizations in the present study are unlikely to be accounted for by other mislocalization phenomena. We suggest that the present audiovisual mislocalization is specific to our complex stimulus configuration, which involves objects on opposing motion trajectories. The present study contributes to a better understanding of audiovisual interactions in cluttered multisensory environments, beyond simple one-to-one interactions between auditory and visual signals.
